# Extragastrointestinal stromal tumor presenting as a scrotal mass: First report from India

**DOI:** 10.1016/j.ijscr.2020.05.079

**Published:** 2020-06-09

**Authors:** Qutubuddin Ali, Krishnanand Anand, Vishal Bansal

**Affiliations:** Department of General Surgery, L.N. Medical College and J.K. Hospital, Bhopal, India

**Keywords:** EGIST, Gastrointestinal stromal tumor, CD34, CD117

## Abstract

**Introduction:**

Gastrointestinal Stromal Tumours (GIST) are the most common mesenchymal tumours that arises from the wall of the gastrointestinal tract. Similar tumours elsewhere in the abdomen are called extra gastrointestinal stromal tumor (EGIST).

**Presentation of case:**

A 68 years male presented with a gradually enlarging scrotal mass over a period of 6month duration. On examination a mass was felt (5 cm × 4 cm) occupying left side of scrotum and abutting the lower pole of left testis. Left inguinal orchidectomy along with excision of mass was done. HPE revealed a spindle cell pattern with low cellularity and Immunohistochemical analysis revealed the tumor reactive for CD 117 and CD 34 which is suggestive of GIST.

**Discussion:**

EGIST have been reported in unusual locations, including the pancreas, prostate, and abdominal wall. The occurrence of EGIST is extremely rare and little is known about their actual origin. The diagnosis is by the demonstration of a specific marker CD 117 and CD34. We describe an unusual case of extra gastrointestinal stromal tumor (EGIST) presenting as a primary scrotal mass.

**Conclusion:**

To the best of our knowledge, this is the first Indian case of an EGIST involving the scrotum as primary site and second such case worldwide.

## Highlights

• Gastrointestinal Stromal tumours (GIST) are the most common mesenchymal tumors that arises from the wall of the gastrointestinal tract.
• Similar tumours elsewhere in the abdomen are called extra gastrointestinal stromal tumor (EGIST).
• We describe an unusual case of EGIST presenting as a primary scrotal mass.
• Left inguinal orchidectomy along with excision of mass was done.
• HPE revealed a spindle cell pattern with low cellularity and IHC analysis revealed the tumor reactive for CD117 and CD34 which is suggestive of GIST.

## 1 Introduction

Gastrointestinal stromal tumors (GIST) are the most common mesenchymal tumors that arise from the wall of the gastrointestinal tract [1]. Similar tumors in the soft tissue of the abdomen are called Extragastrointestinal stromal tumors (EGIST). We report first case of a left scrotal mass in a 68-year-old man, which was treated with complete surgical resection. Histological assessment showed a spindle cell neoplasm, which was positive for CD 117 and CD 34 on immunohistochemical analysis, consistent with an EGIST. To our knowledge, there has been no report of a primary GIST in the scrotum in India till date. The work has been reported in line with the SCARE criteria [14].

## 2 Case report

**Fig. 1:**
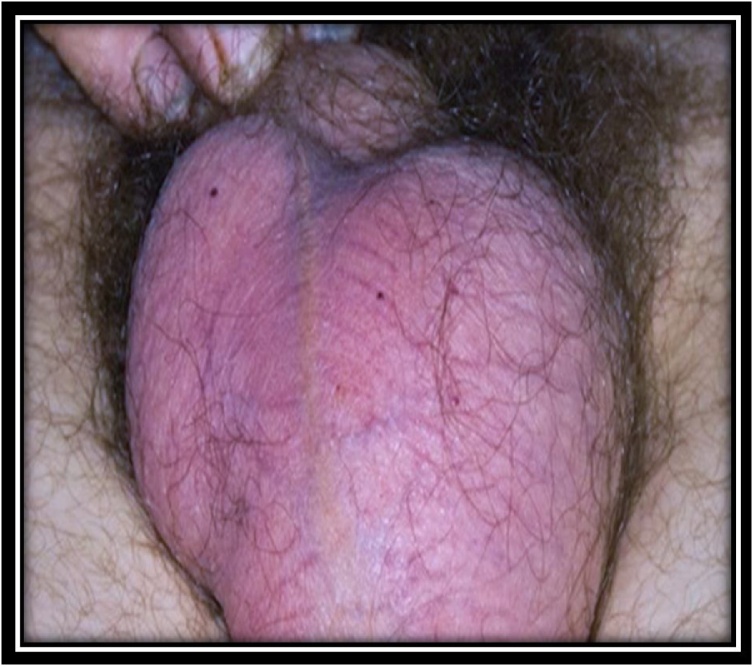
Physical examination revealed a round (a tennis ball), non-tender mass occupying whole the left scrotum.

**Fig. 2:**
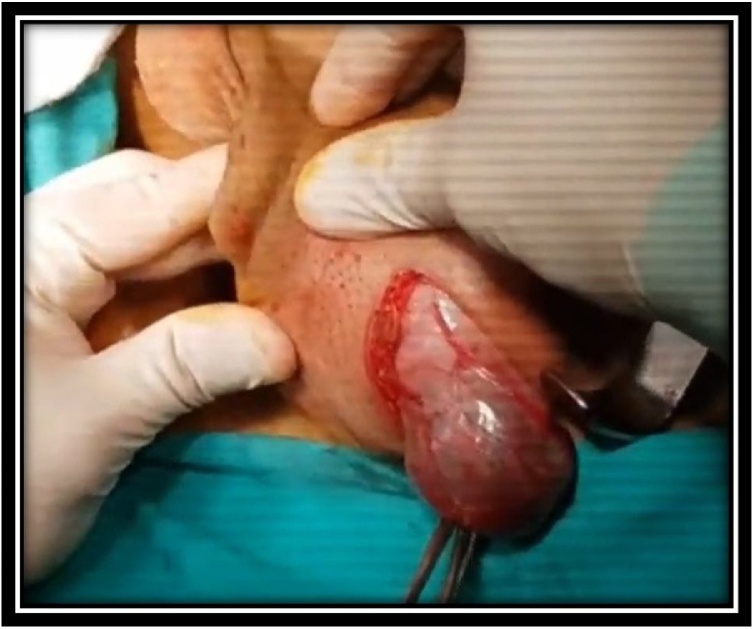
Intra-operative image of tumor.

**Fig. 3:**
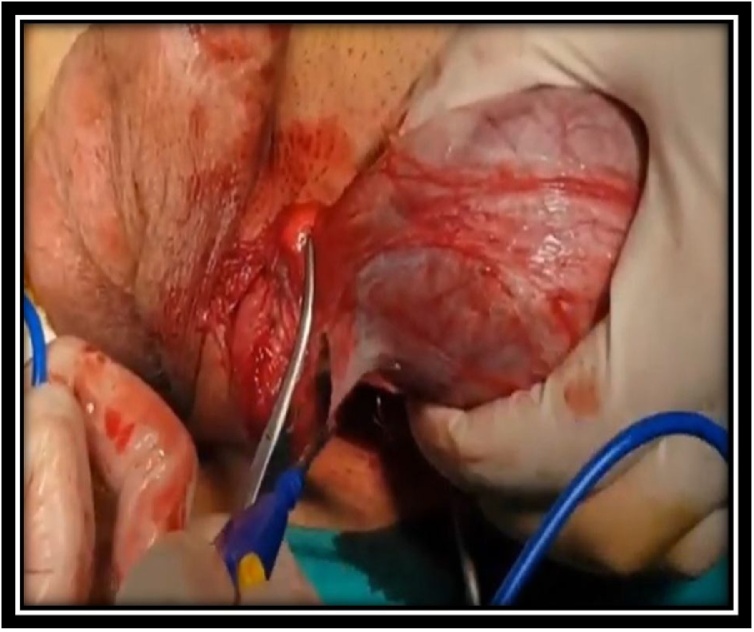
Holding whole of testis along with tumor in hand.

**Fig. 4:**
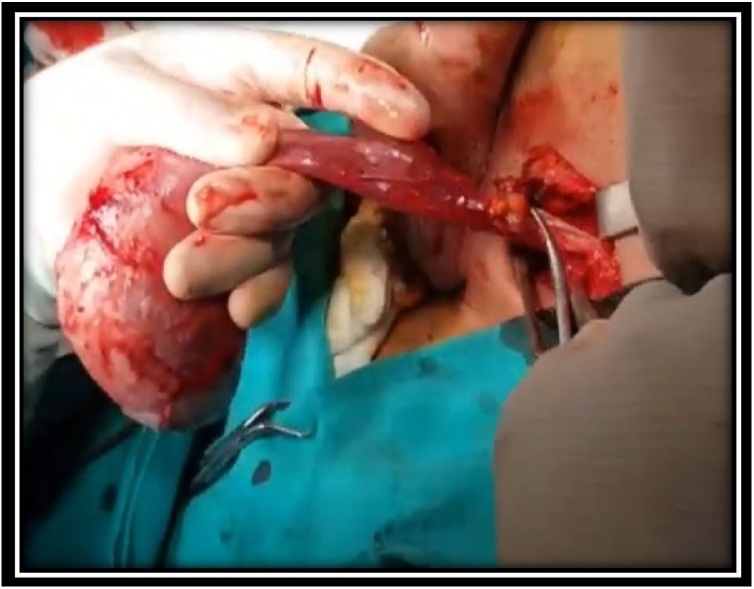
Specimen of high inguinal orchidectomy with spermatic cord.

A 68-year-old man was admitted to our department with chief complaint of a scrotal mass, which had enlarged gradually over a 6 month duration. Physical examination revealed a round, nontender mass, approximate to the size of a tennis ball, occupying the whole left scrotum (Fig. 1). Scrotal USG showed a heterogenous lesion in the left hemiscrotum arising from layer of tunica albuginea. The level of serum tumor markers, including alpha-fetoprotein, human chorionic gonadotropin and lactic acid dehydrogenase, were within the normal range. CT scans of the chest, abdomen did not reveal any lymphadenopathy or mass lesion. There was no evidence of lymph node enlargement or ascites. The patient underwent a high inguinal orchidectomy which was further send for HPE examination (Fig. 2, Fig. 3, Fig. 4).

## 3 Macroscopic and microscopic findings

**Fig. 5:**
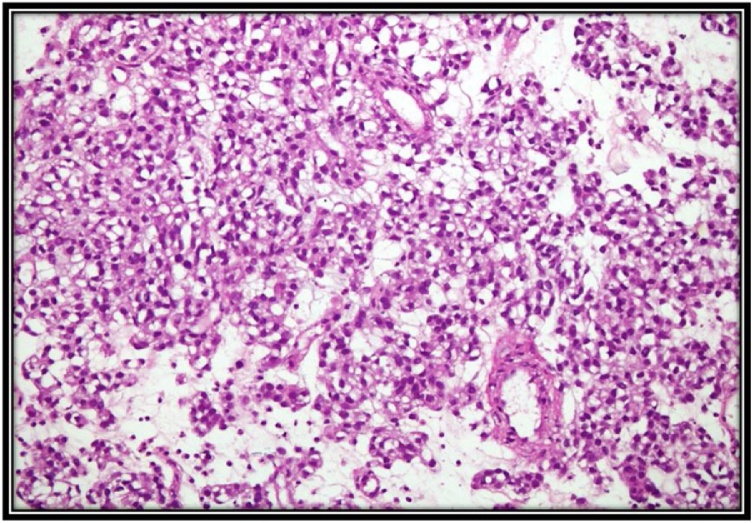
Densely packed spindle cell proliferation.

**Fig. 6:**
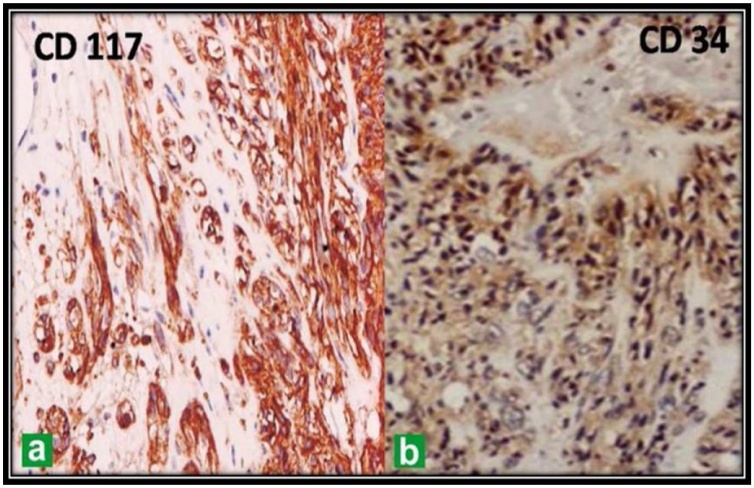
Immunohistochemical stain demonstrates positivity (A): for CD117; (B): for CD34.

Grossly, the surgical specimen consisted of an ovoid, whitish-gray, firm lobulated mass, measuring 6 × 5 × 4 cm and weighing 200gm. It was encapsulated well and cut sections showed yellowish fibrotic lobulated tissue with focal calcification. A 1 × 1 cm cystic change, not necrotic, was noted in the central portion of the mass. Microscopically, the tissue was composed of a densely packed spindle cell proliferation. The cells were predominantly arranged in short, interweaving fascicles with multifocal hyalinization and calcification. Individual cells showed oval, uniform nuclei without any mitosis per 50 high power field and clear cytoplasm. The Ki-67 labeling index was also less than 5%. Immunohistochemical stains demonstrated strong positivity for both **c-kit (CD117) and CD34**. However, the stains were negative for smooth muscle actin, desmin, or the S-100 protein. After an uneventful postoperative course, the patient was discharged 7 days after surgery. There was no recurrence of the tumor during the 3-month follow-up examination (Fig. 5, Fig. 6).

## 4 Discussion

Gastrointestinal stromal tumours are nonepithelial neoplasm that usually arise in the muscular layer of the digestive tract, particularly in the stomach and small intestine. Stromal tumours that arise outside the gastrointestinal tract extremely rare and these lesions are known as EGIST.

**Review of the current published literature on EGIST** has shown that these tumors arise more frequently in the intra-abdominal cavity and retroperitoneum. Also, cases of EGIST have been reported in unusual locations, including the pancreas, prostate, and abdominal wall [5]. The occurrence of EGIST is extremely rare and little is known about their actual origin. Miettinen et al. [7] first reported a series of omental and mesenteric stromal tumours typically positive for CD117, but it was Reithet al. who used for the first time the term extragastrointestinal tumour (EGIST) to describe neoplasms with similar morphological and immunohistochemical characteristics as GISTs, which however occurred in the soft tissue [2].

The usual pathogenesis is a gain of function mutations in the c-KIT gene that result in overexpression of the c-KIT receptor also called CD117. Some of the most essential diagnostic markers, which can distinguish EGISTs from other tumours, such as sarcomas or intra-abdominal desmoid tumours, are c-kit, DOG-1 and PKC [7]. Most studies have shown that the size, mitotic activity and cellularity of EGISTs are the most accurate predictors of an adverse outcome [7]. The findings of previously published studies on EGIST have led authors to believe that these tumors are histologically and immunohistochemically similar tumors, with features that are also similar to those found in GIST, including the expression of *c-KIT* and *PDGFR-*a gene mutations [2,3,4] (Song Zheng, Huang, Tao, & Pan, 2011). A great percentage approx. 80% of EGIST appeared to be due to metastasis from primary GIST. In the treatment of EGIST, complete surgical resection is recommended and adjuvant imatinib is recommended, and a strict follow-up is necessary due to high recurrence rates. There was no recurrence of the tumor during the 3 month follow up examination in our case.

The diagnosis of GIST and their extragastrointestinal variants are now specifically diagnosed by the demonstration of a specific marker profile with expression of CD 117 and CD34 [8]. It was found that most GIST expressed KIT, a receptor tyrosin kinase encoded by protooncogene c-kit. In normal gastrointestinal wall, KIT is expressed by interstitial cells of Cajal (ICC), which are a pacemaker for autonomous gastrointestinal movement. Because both GIST and ICC are double-positive for KIT and CD34, and because familial and multiple GIST appear to develop from diffuse hyperplasia of ICC, GIST are considered to originate from ICC [9].

There is evidence that despite the fact that virtually all stromal tumors express the c-kit receptor, they display various site-specific differences. Most importantly, the behavior of stromal tumors differs by location, and there seems to be a general trend for increasingly aggressive behavior as one proceeds distally along the gastrointestinal tract [10,11]. For example, the minority of GIST located in the stomach have a good prognosis, whereas those in the small intestine have a significantly worse prognosis.

The prognosis of EGIST is not easy to predict, but EGIST are usually presumed to have a more aggressive course resembling small intestinal tumors from point of location. The National Institute of Health consensus conference has proposed a risk classification of GIST based on tumor size and histopathological mitotic count [8]. If the tumor is less than 5 cm and the mitotic count is below 5 per 50 high power field (HPF), the risk of malignancy is considered to be low. Ando et al. [12] report that the presence of mitotic cells and the Ki-67 labeling index are significant predictive factors for malignant GIST. Recently, Reith et al. [2] assess the histopathologic prognostic factors of EGIST. In their report, cellularity, mitotic activity (> 2 per 50 HPF), and necrosis are associated with statistically significant increases in the risk for an adverse outcome. Overall, 5% of patients who had none or one of the above three risk factors developed adverse outcomes (metastasis and/or death from a complication of their tumor), compared with 92% of patients who had two or three risk factors. In the present case of scrotum primary as EGIST, there were no risk factors (low cellularity, absent mitosis per 50 HPF without nuclear pleomorphism and absent necrosis), suggesting good prognosis. The current definitive treatment for GIST is complete surgical resection. Lymphadenectomy is not necessary, because lymph node metastasis of GIST is very rare [12]. Conventional chemotherapy and radiation therapy have been reported to be ineffective in the treatment of GIST. Imatinib (a tyrosine kinase inhibitor) has been confirmed to be an effective treatment against metastatic and unresectable GIST [13]. In the treatment of EGIST, complete surgical resection is recommended; however, the role of imatinib is unclear. In our case, complete surgical resection of the tumor was performed successfully. Imatinib was used which given a good result of no recurrence or spread of tumor to any other area of body.

Although almost two decades have passed since the initial identification of EGISTs by Miettinen et al. [6], there is still a lack of sufficiently large and/or detailed studies which aim to clarify the characteristics, differentiation, and prognosis of these tumors. This study, combined with with other recent studies, may provide further information to address this knowledge gap.

In summary, the current case emphasizes the possibility that this rare tumor can involve the scrotum as a primary site.Most importantly it was first case of India which we are reporting and second such case in world which will further help to diagnose early such cases come across with scrotal swelling.

## Declaration of Competing Interest

No conflicts of interest.

## Sources of funding

No funding for research.

## Ethical approval

Study Is Exempted From Ethnical Approval.

## Consent

Written informed consent was obtained from the patient for publication of this case report and accompanying images.

## Author contribution

1. DR. QUTUBUDDIN ALI (Associate Professor, UROLOGIST) - Study concept.
2. DR. KRISHNANAND ANAND (Professor & HOD) - Data analysis, Reviewer of case report.
3. DR. VISHAL BANSAL (PG 2^ND^ year) - Corresponding author, Data collection,Writing the paper.

## Registration of research studies

NA.

## Guarantor

Dr. Qutubuddin Ali.

## Provenance and peer review

Not commissioned, externally peer-reviewed.
